# Anaphylaxis induced by *Thalassophryne nattereri* venom in mice is an IgE/IgG1-mediated, IL-4-dependent phenomenon

**DOI:** 10.1038/s41598-019-57231-y

**Published:** 2020-01-17

**Authors:** Fernanda Miriane Bruni, Erica Maria Martins Coutinho, Aline Ingrid Andrade-Barros, Lidiane Zito Grund, Monica Lopes-Ferreira, Carla Lima

**Affiliations:** Immunoregulation Unit, Special Laboratory of Applied Toxinology, Butantan Institute, São Paulo, Brazil

**Keywords:** Allergy, Diseases

## Abstract

We hypothesized that beyond the *Thalassophryne nattereri* venoms ability to induce in mice a strong specific-Th2 response with high levels of specific IgE/IgG1, it would be able to trigger anaphylaxis in sensitized individuals. To investigate whether the venom is capable of inducing an allergic reaction in mice and characterize soluble and cellular mediators involved in this process, BALB/c female mice were sensitized intraperitoneally with decreasing-dose of venom at weekly intervals for 4 weeks and challenged by intraperitoneal, oral or epicutaneous routes with venom 2 weeks later. Our data show that sensitized-mice challenged by all routes showed intense symptoms of anaphylaxis, dependent on the anaphylactic IgG1 and IgE antibodies and mast cells. The late-phase reaction developed after initial symptoms was characterized by the influx of eosinophils, dependent on IL-5, IL-17A and eotaxin produced by Th2 cells in inflamed lungs and skin draining lymph-nodes. Using C57BL/6 deficient mice we demonstrated that IL-4 *KO* mice failed to develop anaphylactic symptoms or local Th2 inflammation, producing low levels of IgG1 and increased levels of IgG2a. Together our results demonstrated that the venom of *T. nattereri* has allergenic proteins that can trigger an allergic process, a phenomenon IgE-IgG1 dependent, IL-4-mediated and negatively regulated by IFN-γ.

## Introduction

Few studies have explored the mechanisms underlying allergy disorders triggered by venom from marine/aquatic organisms, other than the study that identified the venoms of anemone and jellyfish from the phylum Cnidaria as causing anaphylaxis in humans^1^.

Fish of toxicological importance are grouped into the venomous category, which present glands specialized in the secretion of venom and a specialized apparatus (spines with canals or canaliculated) for its inoculation^2^. Although accidents caused by these organisms have a low incidence due to their habitat and do not cause mortality in humans, they can cause serious accidents with varying degrees of morbidity for which there is no specific medicine^3^.

**Figure 1:**
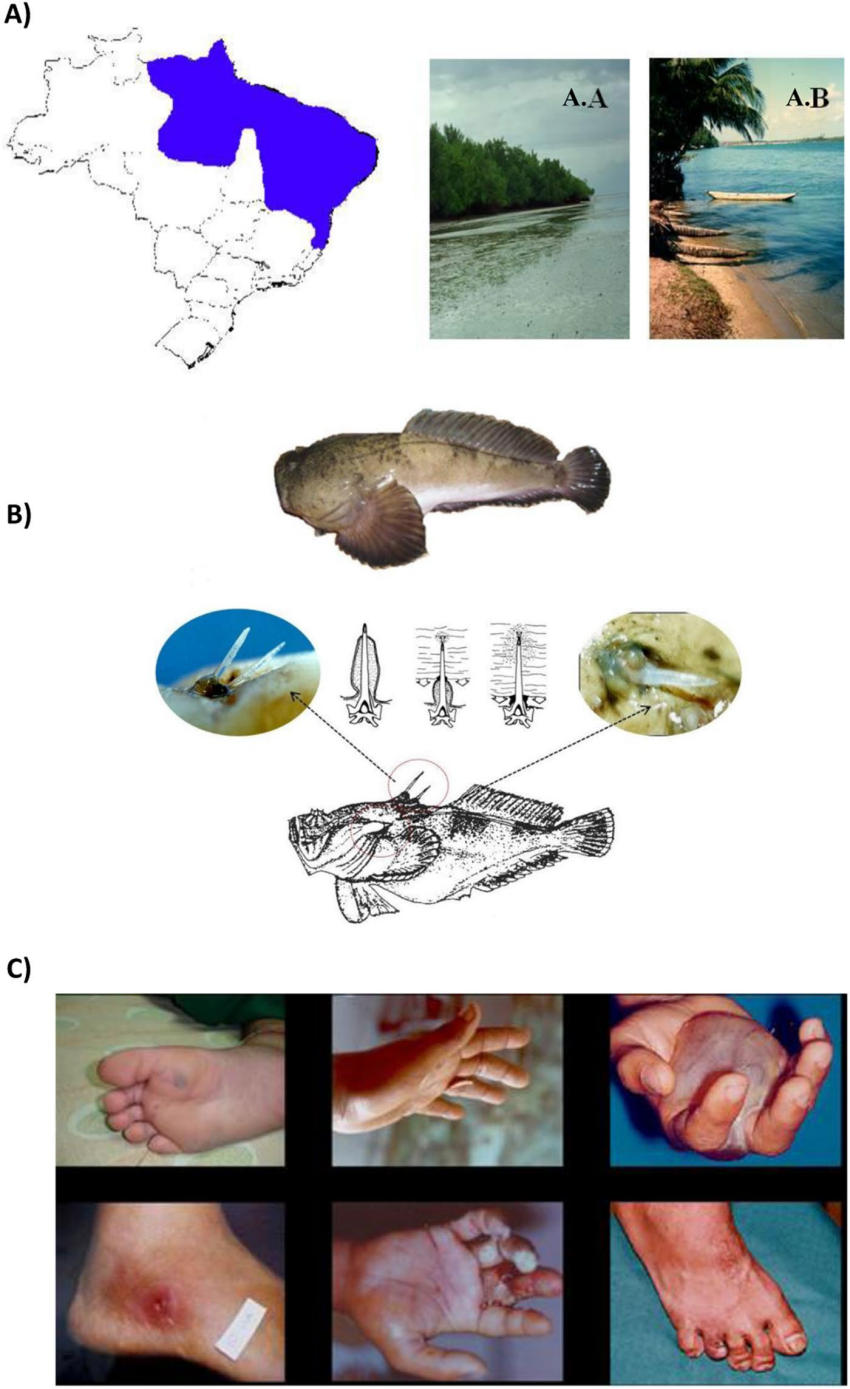
The *T. nattereri* is found predominantly in the states of the North and northeast regions of Brazil (in blue). In places such as Lagoa Mundaú (A.A) and Lagoa do Roteiro (A.B), both in Alagoas. *T. nattereri* has the most complete venom apparatus (**B**), consisting of four stings, two of which are located in the dorsal region (1st segment of the dorsal fin) in the median line, and two laterally placed above the pectoral fin in the opercular region. They all have communication with the venom glands. The inoculation of the venom occurs mainly in the palmar and plantar region and the injury in patients is characterized by pain, edema and necrosis difficult to heal (**C**). All images were taken by the co-author Dr. Monica Lopes-Ferreira.

A growing number of accidents by the *Thalassophryne nattereri* toadfish has been reported among fishermen and bathers in the Brazilian coast (Fig. 1): Salvador^4^, Alagoas^5^, Fortaleza^6^, Natal and Para^7^; and toxicological, biochemical and pharmacological studies have been carried out by our group since 1998^8^. The venom apparatus of *T. nattereri* is composed of two dorsal and two lateral canaliculated spines covered by a membrane connected to venom glands at the base of the fins (Fig. 1B). When the spine penetrates the tissue of victims, the integumentary sheath enclosing the gland press out the venom into a duct and the venom is injected into the victim. According to Fonseca and Lopes-Ferreira^4^, the palm of the hands and the soles of the feet are the most commonly areas affected in humans (Fig. 1C).

The *T. nattereri* venom (V*Tn*) causes pain, edema, erythema and necrosis that remain for a long time without an efficient healing process^9^. In addition, patients that suffer successive attacks have high IgG antibody (Ab) levels up to 6 months after injury^10^. As affected individuals of fishing communities tend to be poor and devoid of the necessary information and financial support to access health care, many cases go unreported. The envenoming represents a great cost to North and Northeast Brazilian communities in terms of public health (http://www.saude.gov.br; http://portalsinan.saude.gov.br).

Lopes-Ferreira *et al*.^11^ evidenced that low dose of venom (0.3 μg/mice), in injured tissue of mice, lead to an intense vascular congestion, stasis of blood flow in postcapillary venules and capillaries, beyond focal transient constrictions in arterioles. Curiously, low numbers of phagocytic cells during the first 24 h after V*Tn* injection and the presence of necrotic material which had not been cleared out seven days after envenomation were described in injured tissues^12^. In addition, we observed a delayed influx of neutrophils to the injured site of venom-injected mice, arriving only after 24 h^13^.

The reproduction of *T. nattereri* envenoming in mice not only demonstrated a strong specific-Th2 immune response^14,15^, but also a long-lasting humoral memory response with high levels of specific IgG1 and IgE Abs^16^. Moreover, we showed that Natterins, a family of proteases with kininogenase activity^17^ and the main components in the venom that trigger the toxic effects^18^ are responsible for the sustained Th2 humoral response in mice. Komegae *et al*.^19^ confirmed that the intraperitoneal immunization of BALB/c mice with proteolytically active Natterins induces a polarized Th2 response with elevated titers of anaphylactic specific-IgG1 and -IgE until 120 days. We also found that inactivation of protease activity abolished the ability of the Natterins to induce persistent production of specific-IgG1, total-IgE and mainly the production of anaphylactic Abs as IgG1 and IgE, favoring the development of specific Abs of the IgG2a subtype, characteristic of Th1 response^19^.

Thus, we hypothesized that the toxins of *T. nattereri* venom although immunogenic would also be capable of inducing an allergic process, characterized as chronic and Th2 mediated. Using a series of *in vivo* approaches, we subjected BALB/c female mice to Th2 sensitization after several intraperitoneal injections of decreasing-dose of V*Tn* with adjuvant followed by challenge with venom by different routes: local (peritoneal) or distal (nasal or epicutaneous). Thereafter C57BL/6 sufficient or deficient mice for some cytokines were used to evaluate the contribution of molecular pathways involved in venom hypersensitivity. Together our results show that the sensitized-mice with decreasing dose of venom developed: a) anaphylaxis with scores ranging from mild to severe, depending on challenge routes; b) produced anaphylactic IgG1 and IgE Abs; c) showed Natterins-specific IgG in the sera; d) recruited eosinophils and neutrophils to the lungs and to the skin later after decay of symptoms. The acute phase is triggered by PAF released after Natterins-IgE/IgG1 activation of mast cells; and the later reaction, mediated by IL-4 derived from CD4 T cells and antagonized by IFN-γ.

Our data in mice allow us to suggest that envenomated and consequently sensitized individuals with allergenic proteases of the *T. nattereri* fish venom when re-exposed to the venom can develop symptoms of anaphylaxis with eosinophilic inflammation in the lungs and in the skin, a process IgE/Th2 mediated.

## Results and Discussion

### The venom of *T. nattereri* Brazilian fish triggers anaphylaxis in mice dependent on mast cell derived-molecules

Clinical observations show that fishermen repeatedly injured with *T. nattereri* develop urticariform reactions in their arms and legs accompanied by wheezing and rhinitis (*personal communications*). Experimental data from our group demonstrated that fish venom induces in mice a humoral immune response characterized by elevated levels of IL-5 and IgE Abs. Thus, we hypothesized that the toxins of *T. nattereri* venom although immunogenic would also be capable of inducing an allergic process, characterized as chronic and Th2 mediated.

The aim of this study was to investigate the capacity of the venom of *T. nattereri* to induce an allergic process in mice. We sought to develop a murine model of acute systemic hypersensitivity reaction to identify the involvement of individual cellular components and soluble mediators decisive in the induction of the emergence of anaphylaxis symptoms, and also, the development of the late-phase inflammatory reaction in the skin and respiratory tract.

The development of murine models of anaphylaxis that mimic the physiological and immunological characteristics of human anaphylaxis has facilitated the understanding of the underlying mechanisms of both phases of allergic immune response. The different models developed for different types of allergens differ not only in the type of allergen used, but also in the protocol of sensitization including different doses and in the parameters applied to the evaluation of the sensitization. In particular, some models of allergen sensitization use intraperitoneal via in the presence of adjuvants^20^ or adjuvant-free^21^, oral immunization with the mucosal adjuvant cholera toxin^22,23^, and more, intranasal or epicutaneous sensitization with or without the use of adjuvants^24,25^. Some model requires multiple sensitizations^26^ or a single sensitization^27^.

**Figure 2:**
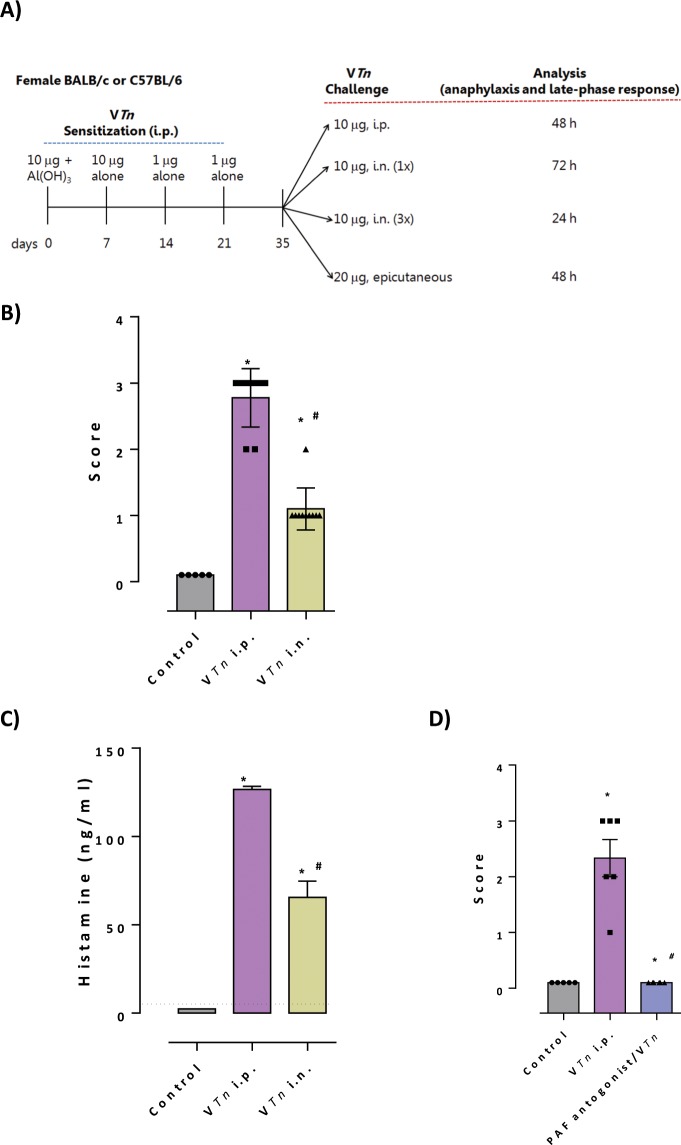
The venom of *T. nattereri* Brazilian fish triggers anaphylaxis in mice dependent on mast cell derived-molecules. Female BALB/c mice immunized by the i.p. injection of 10 μg of venom adsorbed in 1.6 mg of aluminum hydroxide on day 0 were boosted 7 (10 μg), 21 and 28 (1 μg) days later. At day 35, mice were challenged by a single i.p. or i.n. injection or by multiple i.n. instillations. Mice previously shaved and scratched, with hypoallergenic dermal tapes embedded in venom solution (**A**). Twenty-four hours, 48 or 72 h after the last challenge, the symptoms of anaphylaxis were scored (**B**,**D**) and the levels of histamine were measured in the supernatant of peritoneal exudates (**C**). The results represent the mean ± SEM of 5 animals/group. The dashed line represents the detection threshold. *p < 0.05 compared with control-group; ^#^p < 0.05 compared to intraperitoneal challenged-mice.

Here, we subjected BALB/c female mice to Th2 sensitization after repeated intraperitoneal injections of decreasing-dose of V*Tn* with adjuvant (only at day 0) followed by challenge with venom by different routes: local (peritoneal) or distal (nasal or epicutaneous) (Fig. 2A). As demonstrated in Fig. 2B, V*Tn* sensitized-mice challenged by i.p. or i.n. (a single nasal instillation) injections of venom presented clinical symptoms of anaphylaxis within a few minutes after challenge. The continuous licking or scratching of the mouth and ear with paws observed after both challenges (score 1) were followed after 10 min by wheezing and cyanosis around the mouth and the tail in mice challenged by intraperitoneal route (score 3). Control-mice challenged only with saline did not develop symptoms of anaphylaxis.

The symptoms of the acute phase are dependent on mediators released after degranulation of mast cells activated by cross-linking of FcεRI-bound IgE with antigen^28^. Next we figure out the role of the preformed molecules contained in the mast cell granules as histamine, and PAF (platelet-activating factor) the newly synthesized lipid derivative^29^, in the control of anaphylactic symptoms induced by V*Tn*. By both route of challenge, V*Tn* induced the production and release of histamine, especially by intraperitoneal challenge (Fig. 2C). The anaphylactic symptoms induced by V*Tn* were completed inhibited when active PAF receptor was blocked by the selective antagonist ABT-491 (Fig. 2D), but the blocking of PAF receptor did not inhibit the late-phase intraperitoneal inflammation neither the production of anaphylactic IgG1 (data not shown). Together these results allow us to conclude that the *T. nattereri* venom has allergenic proteins capable of triggering IgE/mast cell-mediated anaphylaxis.

### The early-phase response is characterized by anaphylactic Abs production

Sensitization elicits a Th2-cell response, in which IL-4 and IL-13 drive allergen-specific IgE Abs in humans (and IgE and IgG1 in mice) that binding to the FcεRI molecules on a single mast cell. In mice, IgG1 binds only to FcγRIIB and FcγRIII, and produced in a Th2 allergic context in mice is responsible for the passive cutaneous anaphylaxis and systemic anaphylactic response^30–32^. Next, we evaluated the capacity to distinct routes of V*Tn* challenge to induce anaphylactic Abs production in plasma of sensitized-mice by the evaluation of V*Tn*-specific Abs using ELISA or PCA assays.

**Figure 3:**
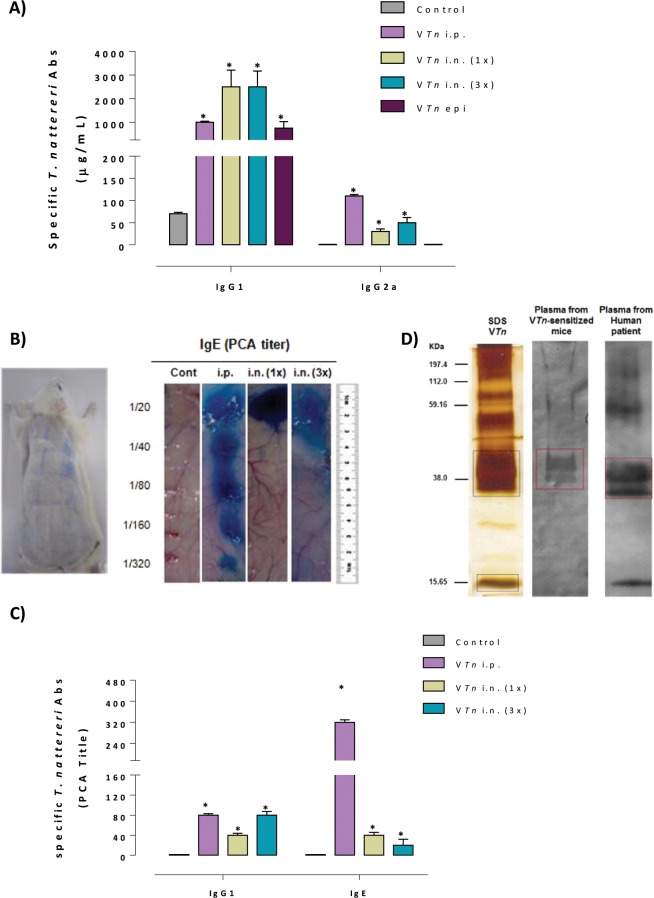
The early-phase response is characterized by anaphylactic Abs production. After the last challenge, the levels of venom specific-IgG1 and IgG2a were determined by ELISA in plasma of BALB/c sensitized-mice (**A**); and the titers of anaphylactic specific V*Tn*-IgG1 in mouse and specific V*Tn*-IgE in rats (**B**) were determined by PCA (**B**,**C**). The PCA titer represents the highest dilution of pooled plasma that gave a positive reaction (diameter >5 mm). V*Tn* was fractionated by SDS-PAGE and proteins were transferred to nitrocellulose membrane that was incubaedt with pool of plasma from allergic mice diluted 1:20 and revealed with goat anti-mouse IgG. Numbers on the left correspond to molecular mass markers (**D**). The results represent the mean ± SEM of 5 animals/group. The dashed line represents the detection threshold. *p < 0.05 compared with control-group.

In Fig. 3 we observe that all route of V*Tn* re-exposition induced in sensitized-mice high concentrations of specific IgG1 and low levels of IgG2a Abs (Fig. 3A). Epicutaneous challenge was ineffective to induce IgG2a Abs production in sensitized mice, producing only specific IgG1. For PCA in mice (IgG1) and in rats (IgE, Fig. 3B), we found that the re-exposition of sensitized-mice to V*Tn* by all route of challenge induced the production of low titers of anaphylactic IgG1 Abs as well as high titer of IgE after i.p. challenge (Fig. 3C). Plasma of mice from the control-group was negative for anaphylactic antibodies.

The allergenic proteases already described in the literature show differences regarding their structure, specificity of the binding site, the class to which they belong and the source of origin. However, there is a consensus that its ability to induce IgE synthesis is related to its proteolytic capacity. Studies in mice confirm that proteolytically active allergens have the ability to induce IgE and IgG1 anaphylactic Abs synthesis that drive allergic response^33–38^. The literature on the protease derived from allergens is extensive, and its role for development of Th2 polarized responses is well established. Next, we confirmed that Natterins were recognized by both sera from *VT*n-sensitized mice and from pool of seven *T. nattereri* human victims (red dotted square, Fig. 3D), demonstrating that after envenoming the production of anaphylactic specific-IgE and IgG1 antibodies is dependent on Natterins.

### The late-phase response induced by *T. nattereri* venom is characterized by leukocytes influx

After the end of the symptoms of the acute anaphylactic reaction and a period without symptoms, allergic individuals present a new inflammatory condition characterized by the accumulation of Th2 lymphocytes and mainly of eosinophils in skin, lung, or nose (depending on the site of challenge), a period denominated as the late-phase response^39^. Then, we evaluated the cellular profile of the late-phase response generated by the *T. nattereri* venom sensitization.

**Figure 4:**
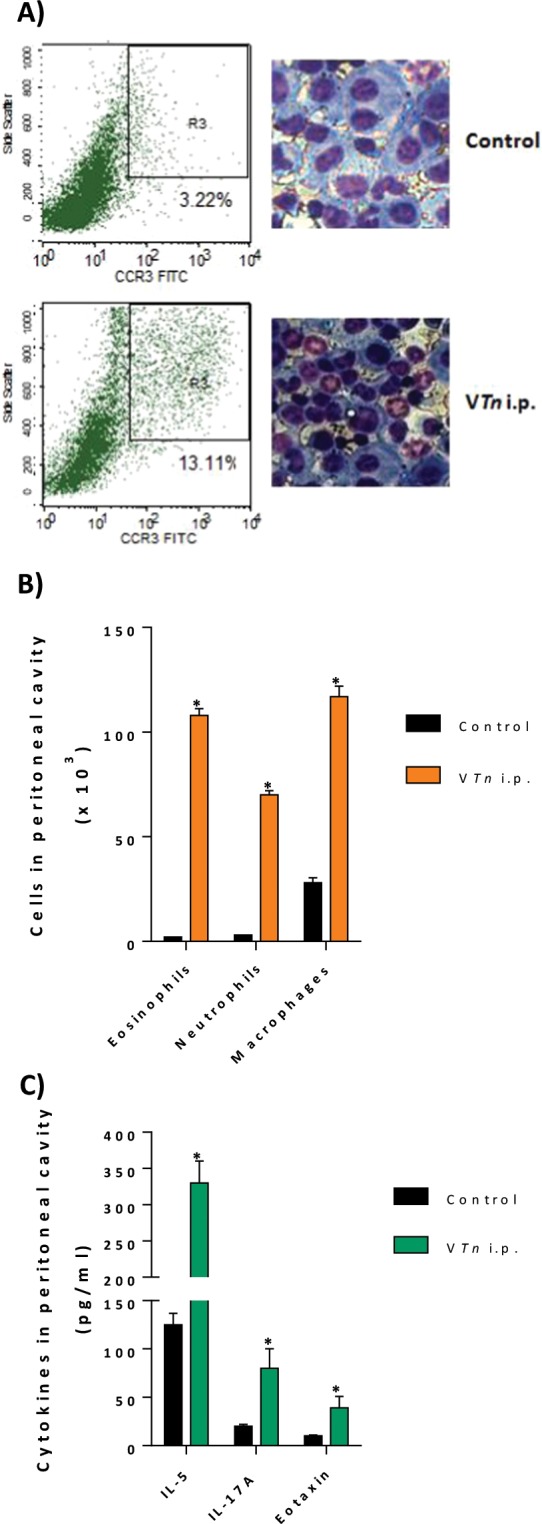
The late-phase response induced by *T. nattereri* venom is characterized by leukocytes influx. After 48 h of intraperitoneal challenge, the influx of eosinophils to exudates of peritoneal cavities of BALB/c sensitized-mice was phenotypically monitored by flow cytometry based on CCR3 membrane expression and morphologically by H&E staining (**A**). The differential cell count (**B**) and the levels of cytokines in exudates of peritoneal cavities (**C**) were also evaluated. The results represent the mean ± SEM of 5 animals/group. *p < 0.05 compared with control-group.

Our results depicted in Fig. 4A show that the V*Tn* intraperitoneal challenge promoted after 48 h in peritoneal cavities of sensitized-mice an accumulation of leukocytes mainly eosinophils as demonstrated by the analysis of CCR3 expression gated cells or by photomicrographs of slides with H&E stained peritoneal cells. The leukocyte influx induced into the peritoneal cavities after 35 days of V*Tn* sensitization was characterized by a mixed inflammatory phenotype during the chronic phase with the presence of macrophages (4-fold) and mainly eosinophils (54-fold) and neutrophils (23-fold) (Fig. 4B), recapitulating one of the hallmarks of severe allergic inflammatory diseases, including cellular infiltration by both eosinophils and neutrophils^40,41^. In control-mice, neutrophils and eosinophils were barely detectable, and resident macrophages were the major myeloid cells in the peritoneal cavities.

Next, we evaluated the levels of the important cytokines and chemokines that drive the development of allergic late-phase reaction. These mediators were evaluated in supernatants of peritoneal exudates by cytokine-specific ELISA (Fig. 4C). We observed that i.p. challenge promoted the release of IL-5 (2.6-fold), an important cytokine responsible for promoting the growth, differentiation and maturation of eosinophils in the bone marrow^42^, the release of IL-17A (4-fold), responsible for the recruitment and survival of neutrophils and macrophages^43,44^; and eotaxin (4-fold), which during the course of the inflammatory response, together with IL-5 promotes the rapid expansion of eosinophilic progenitors in the bone marrow and, subsequently, terminal differentiation in mature eosinophils and migration through the bloodstream to the inflamed tissue^45^. No significant levels of the IL-4, IL-13, IL-10 or IFN-γ were detected in the supernatants of peritoneal exudates of i.p. V*Tn* challenged-mice.

Together these results show that sensitization and intraperitoneal challenge with the venom of *T. nattereri* promote an allergic response characterized by mast cell-dependent acute phase of anaphylaxis followed by the influx of eosinophils to the challenge site guided by Th2 cytokines.

### The airway allergic response triggered by venom

**Figure 5:**
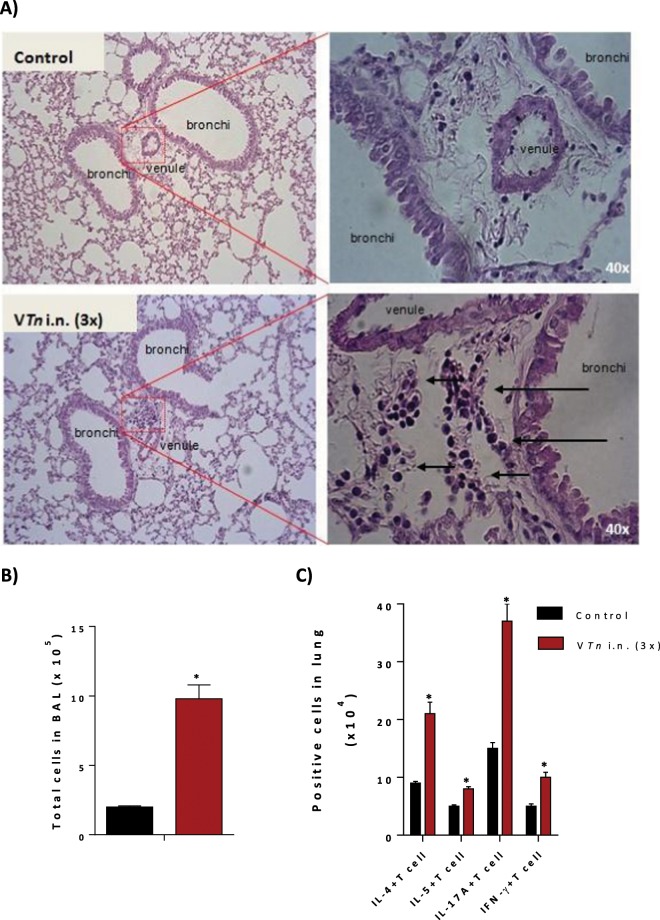
The airway allergic response triggered by venom. Lung sections stained with H&E (**A**) show normal morphology or cellularity in control-mice (*upper*) and perivascular and peribronchiolar eosinophils and neutrophils were observed in BALB/c sensitized-mice (*bottom*). The total number of leukocytes in BAL (**B**) and cytokine-producing CD4 T cells in supernatant of re-stimulated lung cell suspension were determined by flow cytometry (**C**). The results represent the mean ± SEM of 5 animals/group. *p < 0.05 compared with control-group.

Next, we evaluated whether the exposition of intraperitoneal sensitized-mice to nasal instillation of V*Tn* could also triggered an allergic inflammatory response in the lungs as demonstrated by Arizmendi *et al*.^25^. For then, group of i.p. sensitized-mice exposed to 3 consecutive nasal instillations was evaluated after 24 h of the last challenge (Fig. 5).

We observe that the consecutive nasal instillations triggered the passage of a large number of eosinophils through the bloodstream to the pulmonary interstitium accumulating predominantly in the perivascular and peribronchial regions of lung tissues (Fig. 5A), and then to the bronchoalveolar space (almost 5-fold in relation to the control, Fig. 5B). In contrast, lung sections from control-mice showed almost normal histology, with marginal perivascular and peribronchiolar lymphocytic infiltrate (Fig. 5A,B).

By flow cytometry, we confirmed that the consecutive three nasal instillations of V*Tn* in sensitized-mice generated the differentiation with the consequent migration to the lungs of IL-5-producing (2.3-fold), IL-4-producing (1.6-fold), IL-17A-producing (2.5-fold) CD4 T-lymphocytes, as well IFN-γ-producing (2-fold) CD4 T-lymphocytes, compared to control-mice (Fig. 5C).

These results confirm that the repeated exposure of the respiratory tract of mice sensitized with *T. nattereri* venom generates a weak anaphylactic response with mild symptoms, but is capable of triggering a late phase of inflammatory reaction in the lungs. The pulmonary eosinophilic inflammation triggered by airway exposure to V*Tn* is also characterized by the influx of CD4 Th2 lymphocytes in the lung.

### The epicutaneous exposure to the VTn triggers inflammation

In addition to a localized reaction in the lungs, atopic individuals may present in response to dermal exposure pruriginous urticariform reactions with edema^24,46,47^. For assessment of the development of an allergic skin response, V*Tn* sensitized-mice by the i.p. route were challenged on the day 35 by the epicutaneous via with the fixation on the back of hypoallergenic dermal tape embedded with venom (Fig. 2A). After 48 h of challenge, mice were bled and killed to obtain blood, skin and draining lymph nodes. It was not possible to quantify the anaphylactic symptoms triggered by the epicutaneous exposure of the venom since for the observation of the lesions mice were kept anesthetized.

**Figure 6:**
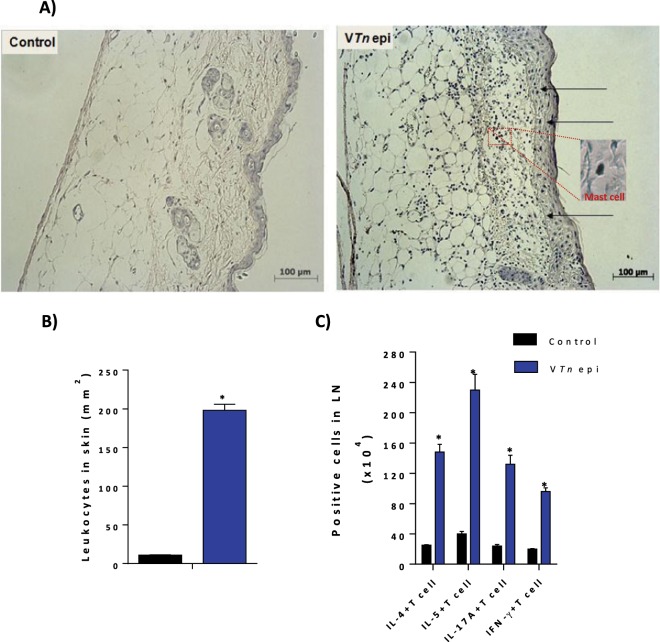
The epicutaneous exposure to the V*Tn* triggers skin inflammation. BALB/c sensitized-mice challenged with epicutaneous exposure had the skin removed for staining with H&E or toluidine blue. Black arrows indicate leukocyte influx into the subcutaneous region of the dermis. Edema and toluidine blue-positive-mast cell (*square*) are observed in venom exposed mice (*right*) in relation to the control-mice (*left*). The total number of leukocytes in draining lymph nodes cell (**B**) and cytokine-producing CD4 T cells in the supernatant of re-stimulated cell suspension were determined by flow cytometry (**C**). The results represent the mean ± SEM of 5 animals/group. *p < 0.05 compared with control-group.

Mast cells are been demonstrated develop a critical role in neurogenic inflammation leading to pain and itch^48,49^. The histological analysis of skin sections stained with H&E and the quantification of the number of cells in a section of 400 mm^2^ per slide revealed a dermatitis with edema (Fig. 6A) and infiltration of inflammatory cells in the subcutaneous region (Fig. 6B), consisting primarily of mononuclear cells, eosinophils, and toluidine blue-positive mast cells as demonstrated in the red square.

In the draining lymph nodes of mice exposed to epicutaneous challenge, we observed the presence of mainly IL-5- and IL-4-producing CD4 T-lymphocytes (6-fold) compared with control-group, followed by IL-17A-producing T cells (5.5-fold) and also by IFN-γ-producing T cells (4.8-fold) (Fig. 6C).

These results confirm that the exposure of the skin of mice sensitized with *T. nattereri* venom triggers dermatitis with edema and subcutaneous infiltration of CD4 Th2 lymphocytes that drive leukocytes influx.

### Modulation of the acute symptoms and the late-phase of inflammatory reaction induced by *T. nattereri* venom

**Figure 7:**
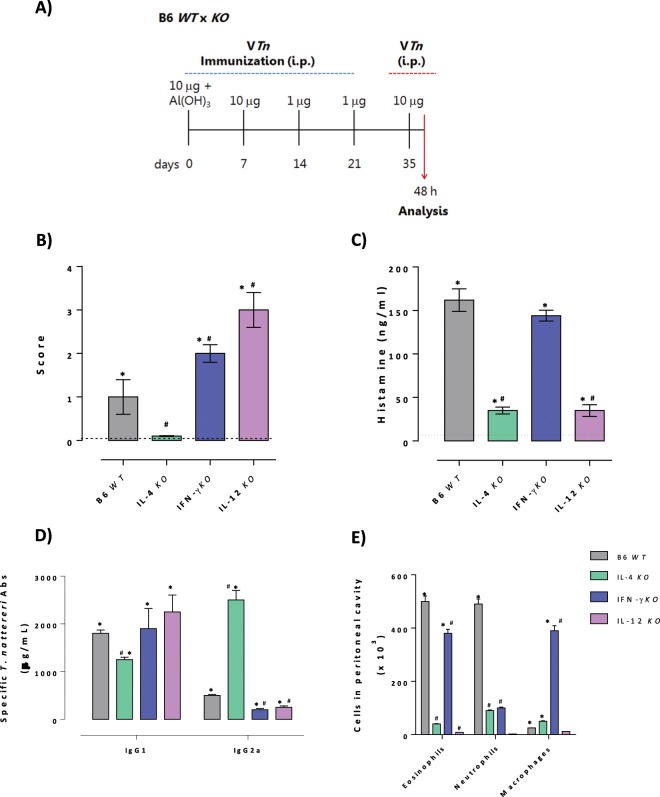
Modulation of the acute symptoms and the late-phase of inflammatory reaction induced by *T. nattereri* venom. Female C57BL/6 *WT* or IL-4 *KO*, IFN-γ *KO* or IL-12 *KO* i.p. sensitized-mice were challenge by i.p. route and 48 h later (**A**) the symptoms of anaphylaxis were scored (**B**), the levels of histamine were measured in the supernatant of peritoneal exudates (**C**); and the plasmatic levels of venom specific-IgG1 and IgG2a were determined by ELISA and PCA (**D**). The PCA titer represents the highest dilution of pooled plasma that gave a positive reaction (diameter >5 mm). The total number of leukocytes in exudates of peritoneal cavities was also evaluated (**E**). The results represent the mean ± SEM of 5 animals/group. The dashed line represents the detection threshold. *p < 0.05 com***p***ared with control-group; ^#^p < 0.05 compared to V*Tn*-challenged-*WT* mice.

The biphasic hypersensitivity reaction is a systemic disorder dependent on an antigen-specific IgE and cytokines as IL-3, IL-4, IL-5, IL-9 and IL-13 produced by Th2 cells. To confirm the role of these cytokines in the development and maintenance of the allergic process induced by the V*Tn*, C57BL/6 IL-4-, IFN-γ- or IL-12-deficient mice were used for sensitization and challenge by intraperitoneal route (Fig. 7A).

In the Fig. 7B we observed that the challenge with the venom of sensitized C57BL/6 *WT* mice triggered the onset of anaphylactic symptoms of score 1, compared to the normal control-mice. Deficiency in the IL-4 in mice abolished the ability of the venom to induce mild anaphylactic symptoms, but in contrast, mice deficient in the IFN-γ or IL-12 had an increased anaphylactic symptoms with score 2 and score 3, respectively. Next, the supernatants of peritoneal exudates of *WT* or *KO* mice were evaluated for histamine (Fig. 7C). After i.p. challenge of *WT* sensitized-mice we notice the presence of high levels of histamine in response to V*Tn* challenge compared with control-mice (dotted line). However, after i.p. V*Tn* challenge of IL-4 *KO* or IL-12 *KO* sensitized-mice the levels of histamine reduced 2-times compared to *WT* V*Tn* sensitized and challenged-mice. In contrast, no alteration in the level of histamine was observed in IFN-γ *KO* mice (*WT*: 162 ± 13 vs *KO*: 144 ± 6.2). Together these results show that the mild symptoms of anaphylaxis in C57BL/6 induced by V*Tn* are positively regulated by IL-4 and IL-12, and negatively regulated by IFN-γ.

IL-4 produced by Th2 lymphocytes modulates various characteristics of the allergic response such as eosinophilia, mast cells, as well as the production of IgG1 and IgE Abs^50,51^. In another way, IFN-γ modulation of IgG2a production is the hallmark of the Th1 humoral response^52^. Our results presented in Fig. 7D show that C57BL/6 *WT* V*Tn*-sensitized and challenged mice produced a robust IgG1 specific response and low levels of IgG2a. However, the deficiency of IL-4 resulted in slight decrease of IgG1 production and instead enhanced IgG2a production. The absence of IFN-γ did not alter the IgG1 production, but drastically decreased IgG2a synthesis, compared to the V*Tn* challenged C57BL/6 *WT* mice. Also, IL-12 deficiency did not alter the large amount of IgG1 induced by venom and promoted a decrease in IgG2a synthesis. Together, we observe that IL-4 positively regulates IgG1 Abs production and the positive control in the synthesis of IgG2a is coordinated by IFN-γ and IL-12.

Finally, the results presented in Fig. 7E show that the significant recruitment of leukocytes into the peritoneal cavity of V*Tn* sensitized and challenged-*WT* mice was characterized by the influx of few macrophages and mainly high number of eosinophils and neutrophils. However, the functional absence of IL-4 or IL-12 promoted a strong decrease in eosinophils and neutrophils into the peritoneal cavity of the *KO* mice challenged with the venom and, in contrast, the absence of IFN-γ promoted a partial reduction in the influx of eosinophils and strong reduction in the number of neutrophils recruited to the peritoneal cavities in response to venom.

The positive regulation by IL-4 and IL-12 of the mixed inflammatory phenotype during the chronic phase of *VT*n response is corroborated by others that show Th1 cytokines as coadjuvants in the maintenance and exacerbation of allergic manifestations^53,54^. A positive correlation was found between the high levels of IL-12 and the state of activation of Th2 lymphocytes in atopic children with dermatitis^55^. Moreover, Zhang and co-workers^56^ found IL-12 is able to induce IL-4 synthesis by mast cells via Akt/ERK.

This is the first study that describes the induction of anaphylactic reaction by *Thalassophryne nattereri* toadfish venom accompanied by a detailed characterization of the various cellular and soluble mediators involved in this process. Our results allow us to conclude that the venom has allergenic proteins likely Natterins capable of triggering the allergic process in mice, characterized by IgE-mediated anaphylaxis and Th2 cytokine dependent late-phase eosinophilic inflammation. In addition, our data confirm that *T. nattereri* venom-induced anaphylaxis in sensitized-mice is an IgE/IgG1-mediated, IL-4-dependent phenomenon produced by allergen-specific Th2 lymphocytes and negatively regulated by IFN-γ. Finally, we observed a positive participation of IL-12 in the induction of localized inflammatory exacerbation, controlling together with IL-4 the influx of eosinophils and neutrophils.

The establishment of a murine model of anaphylaxis induced by *T. nattereri* fish venom represents a progress, since elucidates the cellular and molecular mechanisms of the allergic reaction and becomes a valid proof for proposing therapeutic intervention to prevent and treat allergic episodes in human victims.

## Material and Methods

### Mice

The study with mice and rats was carried out accordance with the recommendations in the Guide for the Care and Use of Laboratory Animals of the Brazilian College of Animal Experimentation and was approved by the Committee on the Ethics of Animal Experiments of the Butantan Institute (Permit Number: 552/08) and of University of São Paulo (Permit Number: 60/70/2). All surgery was performed under sodium pentobarbital anesthesia, and all efforts were made to minimize suffering. Female BALB/c mice (5–6 weeks old) were obtained from a colony at the Butantan Institute, São Paulo, Brazil. Female C57BL/6 wild type (*WT*), C57BL/6 IL-4 knockout (*KO*), IFN-γ *KO*, and IL-12 *KO* mice were obtained from a colony at Institute of Biomedical Sciences II, University of Sao Paulo, Sao Paulo, Brazil. Animals were housed in a laminar flow holding unit (Gelman Sciences, Sydney, Australia) in autoclaved cages on autoclaved bedding, in an air-conditioned room in a 12 h light/dark cycle. Irradiated food and acidified water were provided *ad libitum*.

### Thalassophryne nattereri venom

All necessary permits for *T. nattereri* capture, conservation and venom collection were approved by the IBAMA (*Instituto Brasileiro do Meio Ambiente e dos Recursos Naturais Renováveis*, Permit Number: 14693-1). *T. nattereri* fish venom (V*Tn*) was obtained from fresh captured specimens at the Mundau Lake in Alagoas, state of Brazil with a trawl net from the muddy bottom of lake. No protected specimens were captured and fish were transported to Immunoregulation Unit of Butantan Institute. Venom was immediately extracted from the openings at the tip of the spines by applying pressure at their bases. After centrifugation, venom was pooled and stored at −80 °C before use. Endotoxin content was evaluated (resulting in a total dose <0.8 pg/mL LPS) with QCL-1000 chromogenic *Limulus amoebocyte* lysate assay (Bio-Whittaker) according to the manufacturer’s instructions. After that fish were anesthetized with 2-phenoxyethanol prior to sacrifice by decapitation.

### Sensitization and challenge by *T. nattereri* venom

Groups of 5 mice were immunized with intraperitoneal (i.p.) injections of 10 µg of V*Tn* plus 1.6 mg of aluminum hydroxide (Al(OH)_3,_ Pepsamar, Sanofi-Synthelabo SA, Rio de Janeiro, Brazil) in 500 µL of PBS on day 0, and boosted only with V*Tn* on days 7 (10 µg in 500 µL), 14 (1 µg in 500 µL) and 21 (1 µg in 500 µL). Mice injected only with adjuvant were considered as the control-group. At day 35, independent group of mice were challenged with V*Tn* by intraperitoneal (10 µg in 500 µl), intranasal once or three time (i.n., 10 µg in 50 µl), or by epicutaneous route in anesthetized and previously shaved mice (epi, hypoallergenic dermal tape soaked with 20 µg in 200 µl was adhered to the scratched back). The low dose of V*Tn* used here (total of 32 μg) was adjusted to prevent death induced by the LD_50_ of 91 μg per animal according to Lopes-Ferreira *et al*.^57^, and to minimize the extreme discomfort as strong intercostal stretching and shivering hair.

After 48, 72, 24, or 48 hours respectively, blood samples were collected by retroorbital bleeding and mice were killed by the injection of lethal dose of sodium pentobarbital anesthesia, and peritoneal fluid, bronchoalveolar lavage fluid (BAL), lung and skin were harvested at various time points, and single cell suspension was prepared. For evaluation the role of platelet-activating factor (PAF) activity in hypersensitivity induced by V*Tn*, fourteen days after the last immunization and 1 hour before i.p. V*Tn* challenge an independent group of BALB/c sensitized-mice was treated by i.p. injection of ABT-491 at 0.4 mg/Kg (Sigma-Aldrich).

### Assessment of hypersensitivity responses

Symptoms of systemic anaphylaxis induced by V*Tn* appeared within 15 to 30 min and reached a peak at 40 to 50 min thereafter. Symptoms were evaluated using a scoring system modified slightly according to^22^ and scored as follows: 0 = no symptoms; 1 = scratching and rubbing around the nose and head; 2 = puffiness around the eyes and mouth, pilar erecti, reduced activity, and/or decreased activity with increased respiratory rate; 3 = wheezing, labored respiration, and cyanosis around the mouth and the tail; 4 = no activity after prodding or tremor and convulsion; and 5 = death.

### Determination of histamine levels

After V*Tn* challenge (i.p. or i.n.) peritoneal and bronchoalveolar lavage fluid were collected and prepared for sample acylation. Histamine levels were determined by using an enzyme immunoassay kit (59221, IBL, Hamburg, Germany) based on the competition principle, as described by the manufacturer. Determinations of sample were achieved by comparing their absorbance with a reference curve prepared with known calibration concentrations. Detection limit was 1.3 ng/mL.

### SDS-PAGE and western blotting

The proteins (10 μg) of *T. nattereri* venom were analyzed by SDS-PAGE 12% under non-reducing conditions. After running, proteins were visualized by staining with Silver. After SDS-PAGE, proteins were identified by transferring the proteins to a nitrocellulose membrane (pore size = 0.2 µm, Schleicher & Schüll, Dassel, Germany) and detecting them by immunostaining with plasma from V*Tn*-sensitized mice (1:20 dilution, pool of all challenged-mice) and from seven *T. nattereri* human victims of both sexes followed by goat peroxidase-labeled anti-mouse or anti-human IgG (H + L) (1:2,000 dilution), respectively as a secondary Abs.

### Titration of IgG1, IgG2a and IgE by ELISA

Plasma were tested for IgG1 or IgG2a Abs using V*Tn*-coated 96-well plates and biotinylated goat anti-mouse IgG1 or IgG2a antiserum. The reactions were developed with streptavidin-horseradish peroxidase complex (Sigma Chemical Co. St. Louis, Mo, USA), O-phenylenediamine (OPD) and H_2_O_2_ and the plates were read at 490 nm on an automated ELISA reader (Spectramax, Molecular Devices). Samples were quantified by comparison with a standard curves.

### Evaluation of anaphylactic IgG1 and IgE by passive cutaneous anaphylaxis (PCA)

The anaphylactic activity of IgG1 was evaluated by PCA reactions in mice previously shaved and injected intradermically (50 µL) with three serial dilutions of plasma (inactivated for 1 h at 56 °C) in each side of the dorsal skin. For IgE titration, PCA reactions were performed in rats using non-inactivated plasma. After 2 or 18 h they were challenged i.v. with 50 or 100 µg of V*Tn* + 0.25% of Evans blue solution. Extravasation of Evans blue dye, due to increased blood vessel permeability during the first 30 min of the PCA reactions is dependent mainly on histamine and serotonin released from activated mast cells. All tests were made in triplicate and PCA titers were expressed as the reciprocal of the highest dilution that gave a lesion of >5 mm in diameter. The detection threshold of the technique was established at 1:5 dilutions.

### Peritoneal cell suspension collection

At time points indicated after V*Tn* challenge, mice were killed, and peritoneal cavity exudates were harvested with 2 × 2.5 ml of cold PBS + 10 mM EDTA for cell suspensions that were centrifuged at 1500 rpm for 10 min at 4 °C. Supernatants were stored at −20 °C for cytokine determination by ELISA; and cell pellets were resuspended in 1 ml of PBS + 0.1% BSA. The total leukocyte count was performed and aliquots containing 100 μl of cell suspension were applied on glass slides, subjected to centrifugation at 1000 rpm for 10 min with Cytospin, stained with kit Diff-Quick Stain Set, and analyzed in an optical microscope a 40× objective. For differential cell counts, 300 leukocytes were classified as mononuclear cells or polymorphonuclear neutrophils/eosinophils and counted, based on staining and morphological characteristics, using a light microscope Axio Imager A1 (Carl Zeiss, Germany) with an AxioCam ICc1 digital camera (Carl Zeiss).

### Quantification of cytokines by ELISA

Cytokines were measured in the supernatants of peritoneal exudates after V*Tn*-challenge by a specific two-site sandwich ELISA using antibody pairs purchased from Pharmingen (BD, San Diego, CA, USA). Binding of biotinylated monoclonal antibodies was detected using streptoavidin-horseradish peroxidase complex (Amersham Int., Amersham, UK) and 2-2′-azino-bis (3-ethylbenz-thiazoline-6-sulfonic acid) (Sigma) in 0.1 M citrate buffer containing hydrogen peroxide. Samples were quantified by comparison with standard curves of recombinant mouse cytokines. Detection limits were 3.9 ng/mL for IL-4, 4.21 ng/mL for IL-5, 1.21 pg/mL for IL-17A, 2.56 ng/mL for IFN-γ, and 1.71 ng/mL for eotaxin.

### BAL fluid and lung tissue collection

After the last challenge with V*Tn*, mice were killed by the injection of lethal dose of sodium pentobarbital anesthesia and the tracheas were cannulated. The airway lumina was washed with 4 × 0.5 mL of Hank’s balanced salt solution (HBSS, Gibco BRL, Grand Island, NY) + 10 mM ethylenediaminetetraacetic acid (EDTA). The resulting BAL fluids were immediately centrifuged at 800 rpm, 4 °C, for 10 min. The supernatant was removed and kept at −20 °C. Three sections of lung were dissociated into single cell suspensions by mechanical disruption in Gentle MACS dissociator (Miltenyi). Cells were isolated and restimulated for 16 h at 37 °C, 5% CO_2_ with a cell stimulation cocktail containing PMA at 20 ng/mL and ionomycin at 1 µM in the presence of brefeldin A and monesin at 10 µg/mL. Subsequently, after washing and fixation, cells as CD4^pos^ T cells were assessed for intracellular content of IFN-γ, IL-17A, IL-4, IL-5 by flow cytometer using a FACSCalibur flow cytometer equipped with CellQuest software (BD Biosciences) and were analyzed using CellQuest Software (Becton-Dickinson, San Jose, CA).

### Histological analysis of lungs

After BAL collection, the lung lobes were washed once with ice-cold HBSS then fixed (10% formaldehyde) and paraffin-embedded. Paraffin-embedded sections (5 μm) were stained with hematoxilin/eosin (H&E) to evaluate general morphology. All slides were examined with light microscopy at a magnification of ×10 or 40 (Axio Imager A1, Carl Zeiss, Germany) calibrated with a reference micrometer slide. For each group of five mice, four stained lung sections from each mouse were analyzed.

### Skin and draining lymph nodes collection

In the epicutaneous challenge-mice, the lymph nodes were collected, cells were isolated and re-stimulated for 16 h at 37 °C, 5% CO_2_ with a cell stimulation cocktail containing PMA at 20 ng/mL and ionomycin at 1 µM in the presence of brefeldin A and monesin at 10 µg/mL. Subsequently, after washing and fixation, cells as CD4^pos^ T cells were assessed for intracellular content of IFN-γ, IL-17A, IL-4, IL-5 by flow cytometer using a FACSCalibur flow cytometer equipped with CellQuest software (BD Biosciences) and were analyzed using CellQuest Software (Becton-Dickinson, San Jose, CA). The skin tissue removed was immediately fixed in 10% buffered formalin. The tissue was then processed and embedded in paraffin. Five-micrometer tissue sections were prepared and stained using H&E and toluidine blue methods. The stained paraffin-embedded sections were examined and qualitatively evaluated using light photomicroscopy for inflammatory cell influx. All slides were examined with light microscopy at a magnification of 10x or 40× (Axio Imager A1; Carl Zeiss) calibrated with a reference micrometer slide. The quantification of the number of cells in a section of 400 mm^2^ per slide was done using Axio Vision Rel 4.8 software.

### Statistical analysis

All values were expressed as mean ± SEM. Parametric data were evaluated using analysis of variance, followed by the Bonferroni test for multiple comparisons. Non-parametric data were assessed using the Mann-Whitney test. Differences were considered statistically significant at *p* < 0.05. The GraphPad Prisma 6 (Graph Pad Software, v6.02, 2013) statistical package was employed.

### Ethical statement

Fish were collected according to the Brazilian Environmental Agency (IBAMA - *Instituto Brasileiro do Meio Ambiente e dos Recursos Naturais Renováveis*) under the license number 14693-1.

Female Balb/c mice (5–6 weeks old) were obtained from a colony at the Butantan Institute, São Paulo, Brazil. Animals were housed in a laminar flow holding unit (Gelman Sciences, Sydney, Australia) on autoclaved bedding, in autoclaved cages, in an air-conditioned room under a 12 h light/dark cycle. Irradiated food and acidified water were provided ad libitum. All procedures involving animals were in accordance with the guidelines provided by the Brazilian College of Animal Experimentation (552/08 and 60/70/02).
